# Correlation coefficient local capping REMD adaptive filtering method for laser interference signal

**DOI:** 10.1371/journal.pone.0261875

**Published:** 2022-01-21

**Authors:** Junfeng Wu, Hanyu Chen, Xu Li, Guohua Kang, Yuangang Lu

**Affiliations:** Key Laboratory of Space Photoelectric Detection and Perception of Ministry of Industry and Information Technology, The College of Astronautics, Nanjing University of Aeronautics and Astronautics, Nanjing, China; Valahia University of Targoviste: Universitatea Valahia din Targoviste, ROMANIA

## Abstract

Considering the issue of noise reduction associated with Laser Doppler Interference (LDI) signal, the paper presented a correlation coefficient local capping robust empirical mode decomposition (REMD) filter algorithm for LDI laser sensor that enables more robust reconstruction of the displacement information from an LDI signal. The performance of the algorithm is studied, and it is shown that the algorithm is capable of removing high-frequency noise. Useful information can be extracted more easily by this method, and the Hilbert phase unwrapping displacement reconstructions method based on this algorithm has been experimentally validated. The experimental results show that the proposed method can improve the frequency separation performance in experiments, and is robust against noise interference.

## 1. Introduction

Empirical mode decomposition (EMD) is a useful tool for decomposing signals into intrinsic mode functions (IMF). EMD, as the first portion of the Hilbert-Huang transform (HHT) introduced by Huang et al. in 1998, is used in analyzing non-linear and non-stationary time series data [1]. These IMF represent the data using oscillating waves with local zero mean. In some sense, the decomposition can be compared with a time-varying filter [2]. Signals are decomposed using band-limited filters with bandwidths that vary in time. The main advantage of EMD compared to other time-frequency tools is that it does not use any predetermined filters or transforms [3]. Hence, the analysis is adaptive in contrast to traditional methods such as wavelets where the basic functions are fixed and Low-pass filtering, which require a priori information of a signal’s frequency characteristics to choose appropriate cutoff frequency [4, 5]. It is therefore a self-contained method that preserves the physical properties in the separate IMFs, explaining why it has been successfully applied in many engineering fields [6–16].

This method has many advantages, but it also has disadvantages From the signal decomposition side, end effects and mode mixing happen and are very common in EMD, and it is very difficult to avoid them [17]. From the signal demodulation side, it has negative frequency problem [18]. The sifting iterations number is directly determined by sifting stopping criterion (SSC), and it is crucial to the EMD performance. A number of criteria have been proposed. Flandrin et al. proposed the predefined value criterion for the EMD [19]. Another criterion is Cauchy type standard deviation (SD) criteria proposed by Huang et al. [1998]. The criterion can be implemented by limiting the size of the SD by twice sifting the results as defined below:

$$SD=\sum_{t=0}^{T}\frac{{\left[h_{k-1}(t)-h_{k}(t)\right]}^{2}}{h_{k-1}^{2}(t)}$$ (1)

A typical value is between 0.2 and 0.3. When the computed SD value lies in the specified range, the sifting process is automatically stopped. These kinds of methods are termed as hard SSC in [20]. The hard criteria requires prior knowledge of the threshold, thus, cannot adapt to signals. A soft sifting stopping criterion is proposed, and it can select the optimal iteration number. This method use mean square (RMS) to define the overall energy of this target signal and Excess Kurtosis (EK) indicator which is the Kurtosis value minus 3 to evaluate the peakedness of one signal. The soft SSC can suppress its mode mixing problem [21]. This adaptive mechanism could stop the sifting process based on the value of the objective function and better performance can be achieved. We will use this robust soft sifting stopping criterion(REMD).

EMD methods combined with correlation coefficient consideration have been studied. EMD based Time Dependent Intrinsic Correlation (TDIC) analysis is applied to consider the correlation between two nonstationary time series [22]. The correlation coefficient between these IMFs was estimated. Rodo and Rodriguez-Aria developed the scale-dependent correlation technique [23]. Although these methods detect the correlation between two nonstationary signals by computing the correlation coefficient in a local sliding window, the main problem is to determine the size of this window. An integrated EMD adaptive threshold denoising method was proposed, and it was showed that a larger correlation coefficient can be offered [24]. A method called center frequency statistical analysis (CFSA) was proposed to determine the number of intrinsic mode function [25]. Compared with maximum center frequency observation (MCFO), correlation coefficient (CC), and normalized mutual information (NMI) methods, CFSA is more robust and accurate. Signal detection based on complete ensemble empirical mode decomposition with adaptive noise and approximate entropy was proposed [26], real IMFs similar to the original signal were selected as final parts through a certain threshold. The problem is that some high-frequency noise signal still has a certain CC value. It is not easy to get focal and informative data, which is happened to be the challenging part of denoising data processing.

Considering these challenges, this paper proposes correlation coefficient local capping with intrinsic mode function (IMF) of empirical mode decomposition (EMD), which over advantage fixed correlation coefficient threshold methods. In this way, it enables reconstructing the signal with more physical relationships. Extensive experiments are conducted to validate the proposed method.

In this paper, we combine REMD with adaptive signal selection by correlation coefficient local capping. The contributions of this paper are summarized as follows:

1. we exploit the characteristics of the correlation coefficient between the empirical modes from the EMD and the original signal to study a new approach to denoising signals.
2. This paper adopts a robust implementation of the soft SSC into sifting process of EMD, and this idea can realize the adaptivity of a sifting process.
3. This paper explores a potential application of REMD to laser interference signal, and displacement is demodulated from the reconstructing signal.

The rest of this paper is organized as follows. A flowchart for the correlation coefficient local capping REMD denoising is provided in section 2. In Section 3, validation of the proposed method is carried out on laser interference signal, and shows a potential ability to demodulation of the laser Doppler interference signal. Finally, Section 4 provides the conclusions of this study.

## 2. Proposed method local capping REMD

Correlation coefficient local capping REMD is implemented as following steps:

1. For any given data x(n), Initialize the algorithm: *j* = 1, initialize residue *r*_0_(*t*) = *x*(*n*).
2. Identify all the local maxima and minima of *r*_*j*−1_(*n*).
3. Compute the upper envelope *U*_*j*_(*n*) and lower envelope *L*_*j*_(*n*) by cubic spline interpolation of local maxima and minima, respectively.
4. Compute the mean of the envelope as $m_{j}(n)=\frac{\left(U_{j}(n)+L_{j}(n)\right)}{2}$.
5. Take the difference between the data and the mean as the proto-IMF: compute the *jth* component *h*_*j*_(*n*) = *r*_*j*−1_(*n*)−*m*_*j*_(*n*).
6. *h*_*j*_(*n*) is processed as *r*_*j*−1_(*t*). Let *h*_*j*0_ = *h*_*j*_(*n*) and *m*_*j*,*k*_(*n*), *k* = 0,1… be the mean envelope of *h*_*jk*_(*n*), then compute *h*_*jk*_(*n*) = *h*_*jk*−1_(*n*)−*m*_*jk*−1_(*n*) until the soft stop criterion is satisfied. The stop criterion used here is described as below:
  Define the objective function
$$f_{jk}=RMS_{jk}+|EK_{jk}|$$ (2)
$$RMS_{jk}=\sqrt{\frac{1}{N_{s}}\sum_{n=1}^{N_{s}}(m_{jk}[n]{)}^{2}}$$ (3)
$$EK_{jk}=\frac{\frac{1}{N_{s}}\sum_{n=1}^{N_{s}}(m_{jk}[n]-{{\bar{m}}_{j})}^{4}}{(\frac{1}{N_{s}}\sum_{n=1}^{N_{s}}(m_{jk}[n]-{\bar{m}}_{j},{)}^{2}{)}^{2}}-3$$ (4)
  Where ${\bar{m}}_{j}$ is the mean of *m*_*jk*_[*n*]. If it meets that: (1) the number of zero points (*N*_*zp*_) and extremal points (*N*_*ep*_) is equal, or the difference between them is less than one; and (2) *f*_*k*−2_ < *f*_*k*−1_ and *f*_*k*−1_ < *f*_*k*_ the sifting process stops and returns the (*k*−2)*th* decomposition results. If not, the sifting process does not stop until the number of iterations reaches the maximum iteration number.
7. Compute the *jth* IMF as *IMF*_*j*_(*t*) = *h*_*j*,*k*_(*n*).
8. Update the residue *r*_*j*_(*n*) = *r*_*j*−1_(*n*)−*IMF*_*j*_(*n*).
9. Increase the sifting index j and repeat steps 2 to 8. The signal reconstruction process *x*(*n*), which involves combining the IMFs formed from the EMD and the residual
$$x(n)=\sum_{j=1}^{N}IMF_{j}(n)+r_{N}(n)$$ (5)
10. Compute the correlation coefficients between the input simulation signal and the generated intrinsic mode *Correlation*(*x*(*n*), *IMF*_*j*_), *j* = 1,2,…*N*, *N* is the number of IMFs. The operator *Correlation*() is defined as:
$$\mathrm{Correlation}(X,Y)=r_{XY}=\frac{{\mathrm{Cov}}_{XY}}{S_{X}S_{Y}}=\frac{\frac{\sum \left(X-\bar{X}\right)(Y-\bar{Y})}{\left(N-1\right)}}{S_{X}S_{Y}}$$ (6)
  Where
$$S_{X}=\sqrt{\frac{\sum {\left(X-\bar{X}\right)}^{2}}{N-1}}$$ (7)
$$S_{Y}=\sqrt{\frac{\sum {\left(Y-\bar{Y}\right)}^{2}}{N-1}}$$ (8)
  and based on selected maximum correlation coefficients, we determine the useful IMFs and discard the noisy mode functions (IMFs). Here we reconstruct the signal as
$$\hat{x}(n)=\sum_{j=k+1}^{m-1}IMF_{j}(n)$$ (9)

Here, *k* and *m* are index numbers of local minimum and maximum of correlation coefficient respectively centered on the extreme value of the correlation coefficient, and this is just like capping on the extreme. We consider that the maximum correlation coefficient has the maximum correlation with the original signal, and the surrounding adjacent decompositions have a higher dependence on the original signal.

11. Demodulate displacement from the denoising signal.

$$Displacement=unwrap(Arctan(\theta (n)))\frac{\lambda }{4\pi }$$ (10)

$$\theta (n)=\arctan\frac{Y(n)}{\hat{x}(n)}$$ (11)

$$Y(n)=Hilbert[\hat{x}(n)]=\frac{1}{\pi }\int_{-\infty }^{+\infty }\frac{\hat{x}(\tau )}{n-\tau }d\tau$$ (12)

Where λ is the wavelength of the laser diode. Unwrap is the function for unwrapping the phase angle. This function corrects the radian phase angles by adding multiples of ±2π when absolute jumps between consecutive phase angle are greater than or equal to the jump tolerance of π radians. Arctan is the inverse tangent.

**Fig 1:**
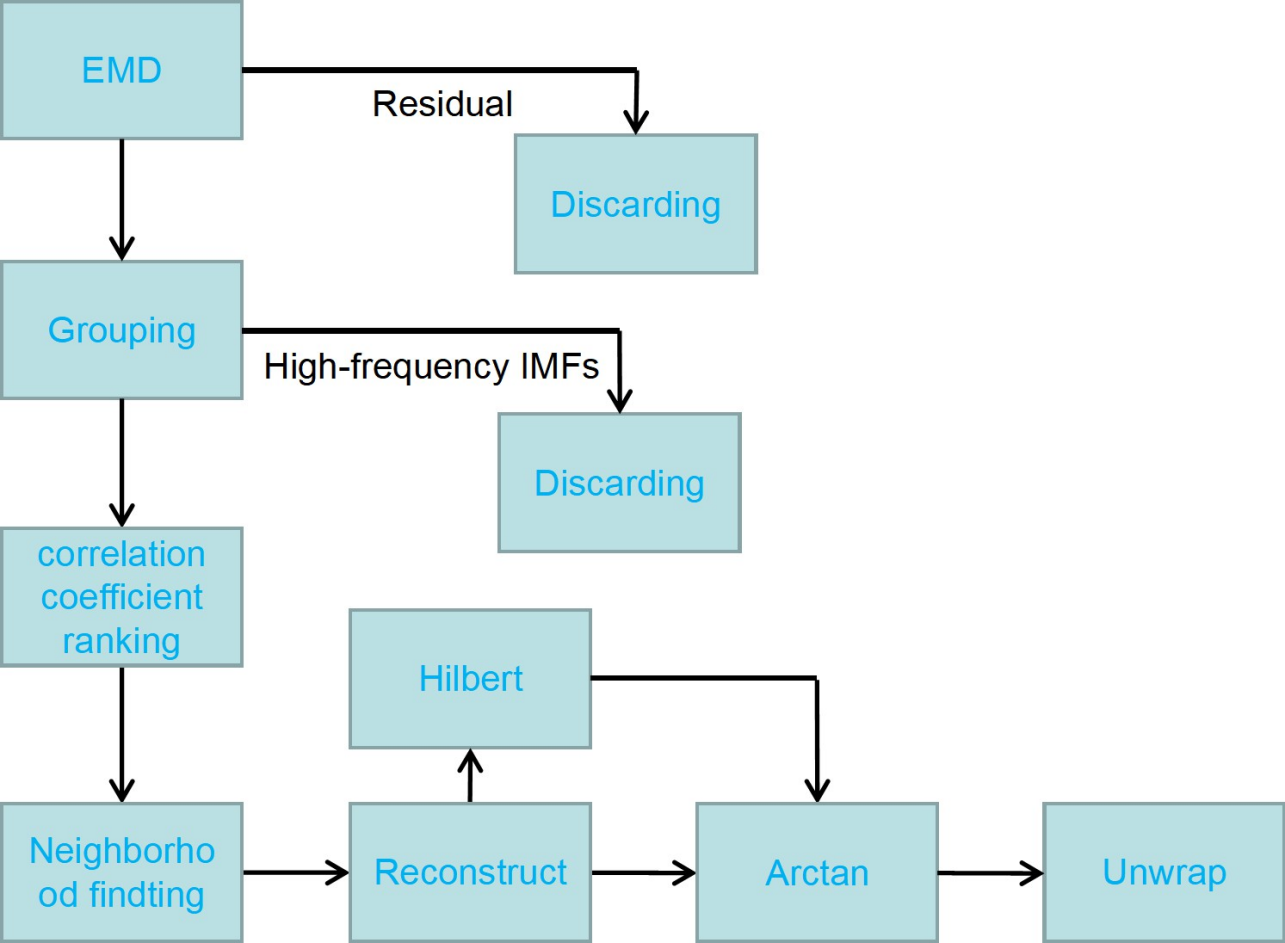
Flowchart of local capping REMD filter.

As shown in the flowchart in Fig 1, the displacement is calculated as follows: target signal → REMD → local capping → reconstruction noise-free signal → Hilbert signal → Arctan signal → unwrapping transform → get displacement.

## 3. Experiment

**Fig 2:**
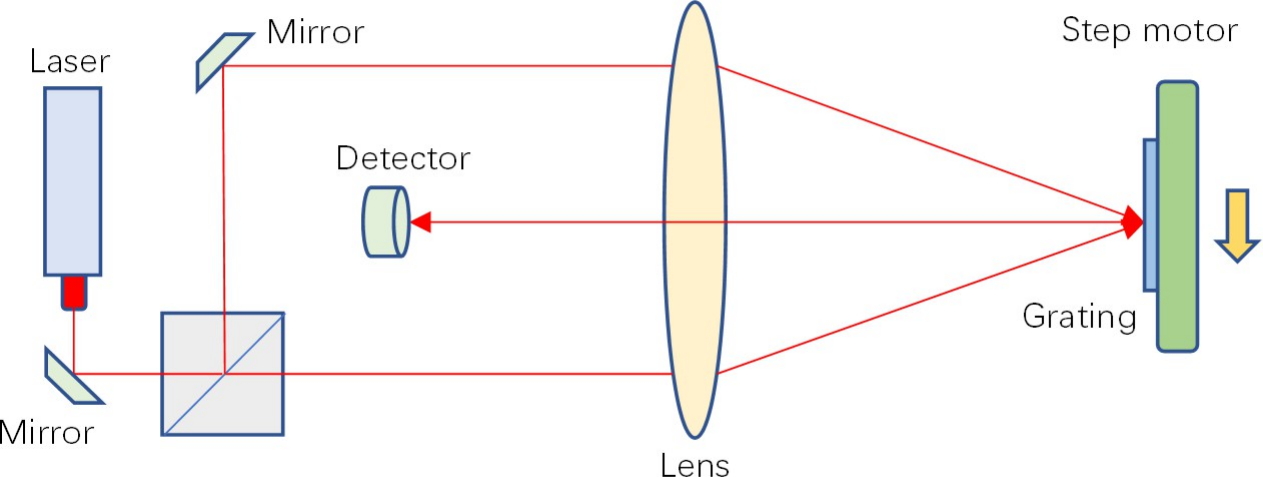
Scheme of the experimental setup.

The validation experiment is carried out according to the following steps. Firstly, the grating is placed on the guide rail driven by the stepping motor. Place a laser interferometer next to it, and scheme of the experimental setup is in the Fig 2. The signal measured by the interferometer is the input signal. Because the laser of the interferometer has non single-mode characteristics, there will be high-frequency interference signals in the measured signals. The motion of the stepper motor has low-frequency characteristics, resulting in the Doppler effect and Doppler frequency shift.

**Fig 3:**
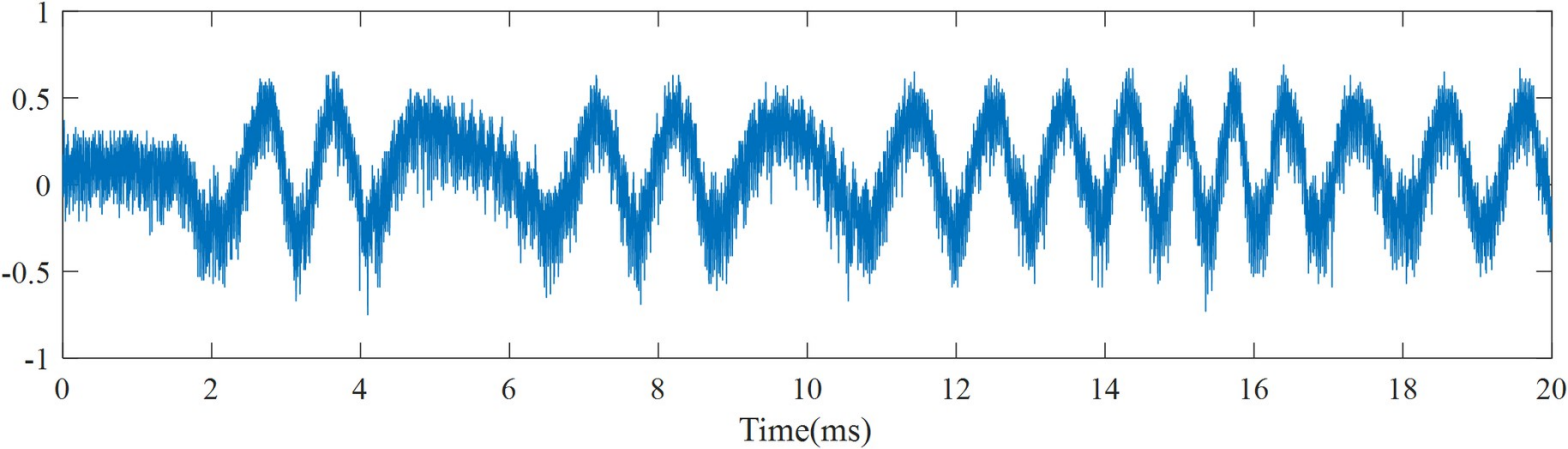
Waveforms of the laser interference signal.

The method mentioned above is applied to this signal as seen in Fig 3 to extract the displacement signal. Remove the noise signal, and then further demodulate the displacement.

**Fig 4:**
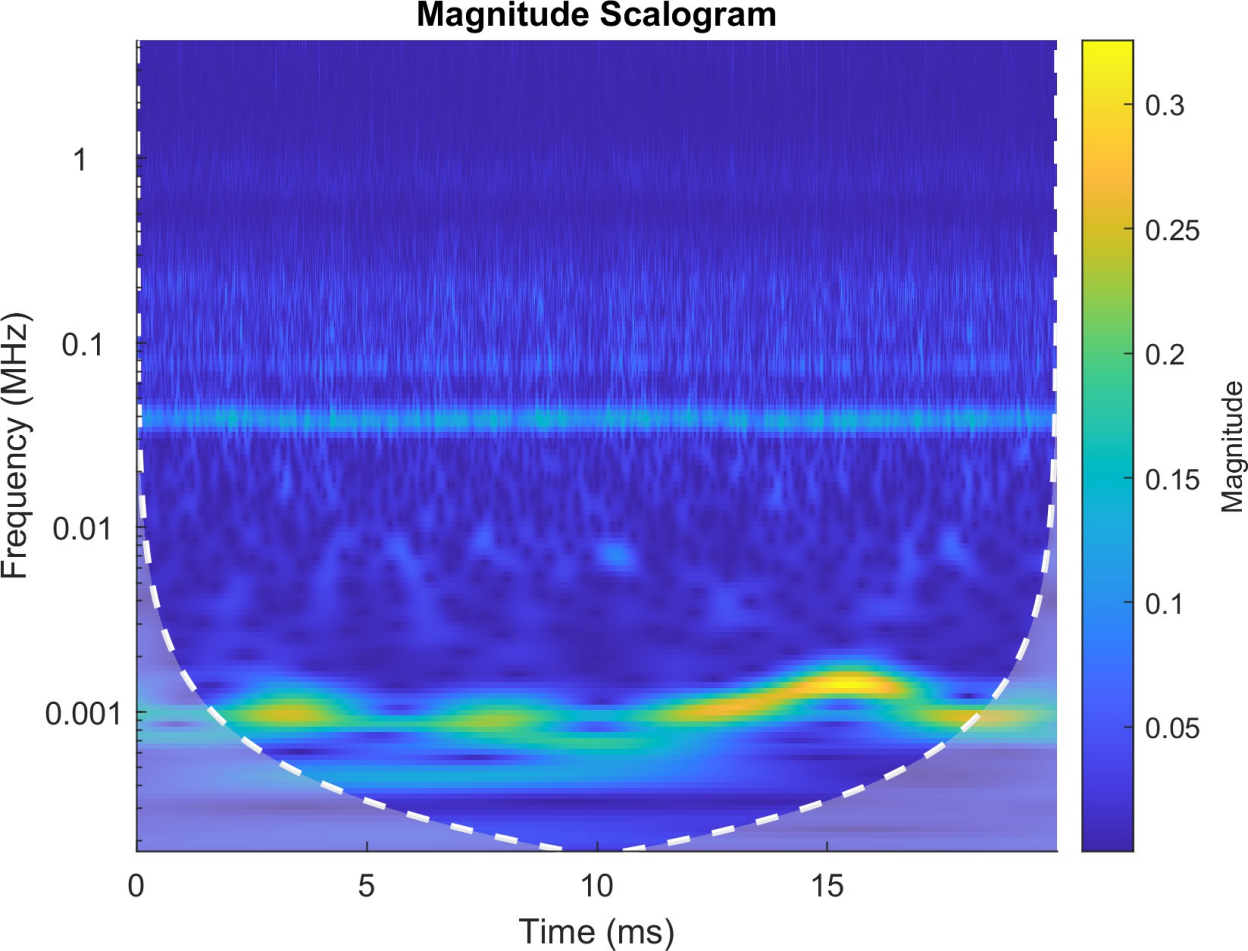
CWT of the input signal.

Firstly, the continuous wavelet transform (CWT) based on Morse wavelet is used to analyze signals jointly in time and frequency. It can localize these transients in addition to characterizing oscillatory components in the signal. A signal about 1000-Hz occurs from 14 milliseconds to 16 milliseconds with the maxima magnitude from the global views, as seen in Fig 4. Additionally, there are two transients at 3.5 and 8 milliseconds, and the whole signal is corrupted by noise.

**Fig 5:**
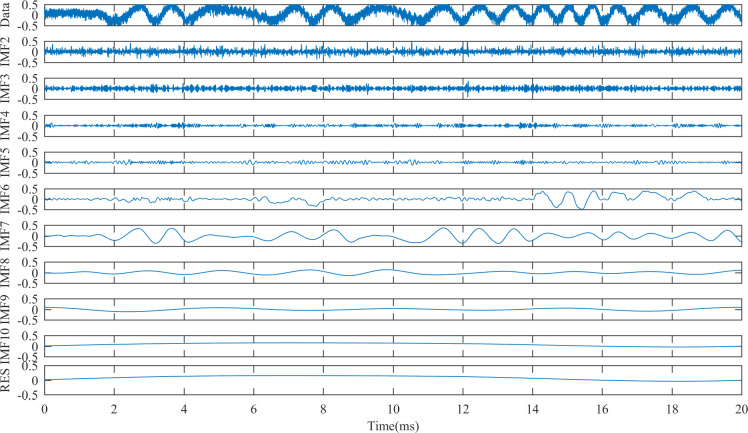
REMD of the input signal.

Then we REMD decompose the input signal, as seen in Fig 5. Through REMD decomposition, nine components and one residue can be obtained. It can be seen that the components of the first five groups are all composed of high-frequency signals. The last four components are composed of low frequencies, and the seventh group has the highest shape similarity with the original signal. In the following calculation, we will use the correlation coefficient to calculate the correlation coefficient between each group and the original signal. It can be seen that the similarity of the shape is also reflected in the size of the correlation coefficient.

**Fig 6:**
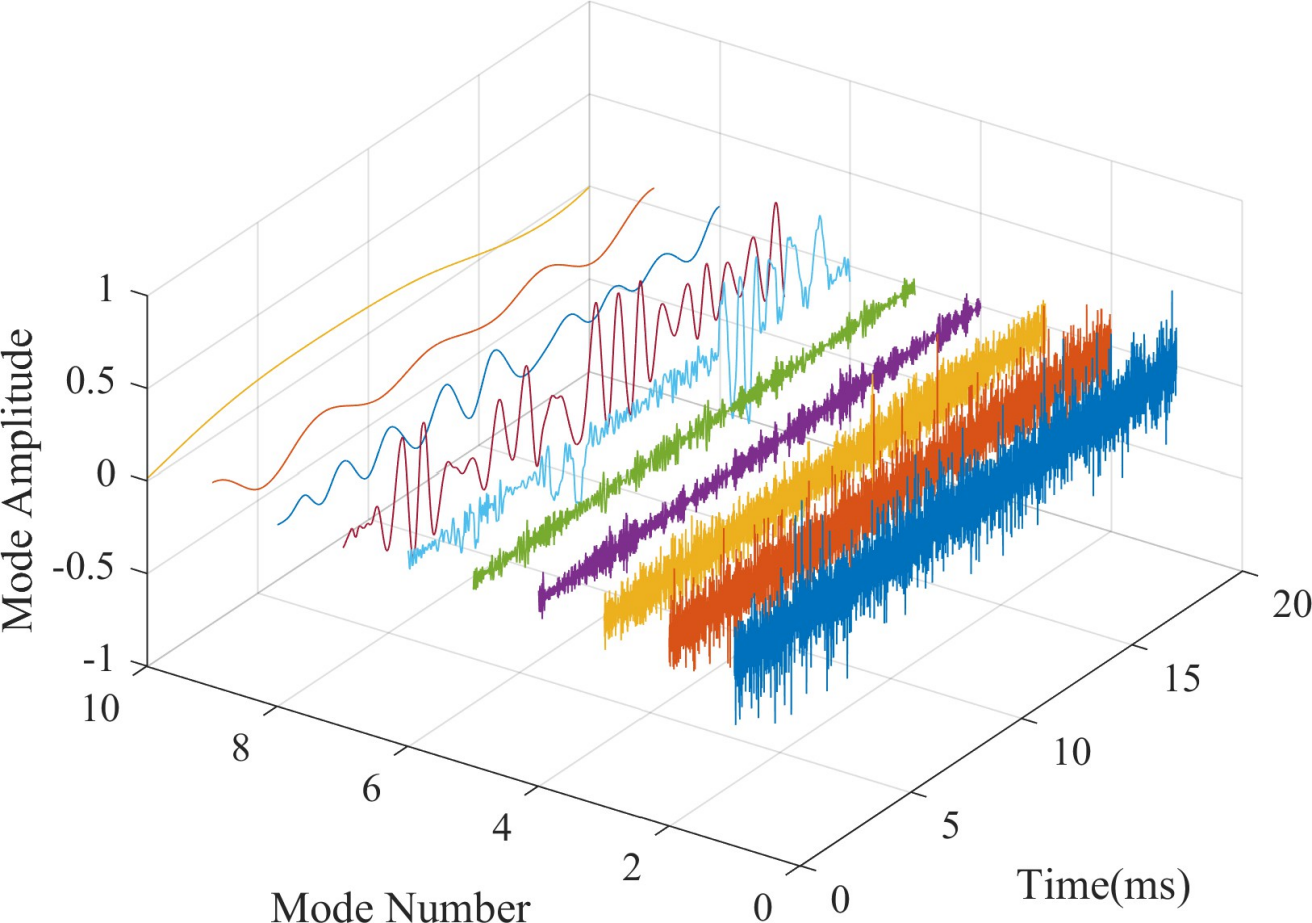
Comparison of IMFs and residual of REMD.

In Fig 6 we put all the components into one picture, which can more clearly compare the differences in frequency and amplitude between them. You can see that the seventh group of signals is highlighted in many components

**Fig 7:**
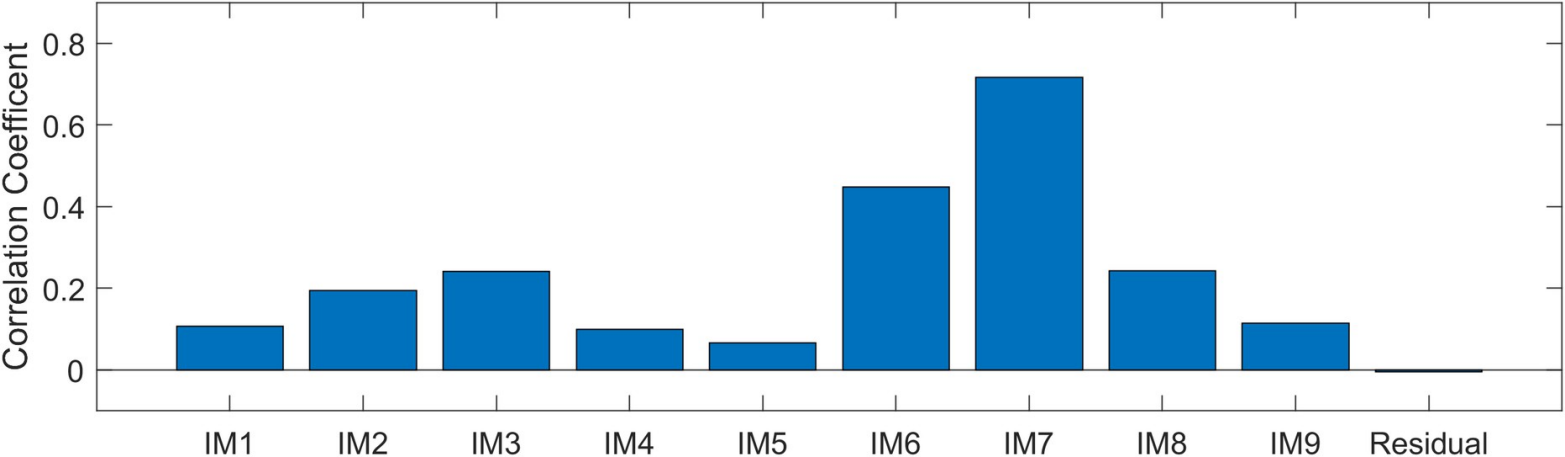
The correlation coefficient of IMFs and residual.

The maximum correlation coefficient corresponds to the optimal value of the shape parameter, and here the maximum correlation coefficient is finally taken as the similarity score between IMFs and the original signal. Fig 7 below shows that the correlation coefficient between IMFs and the original signal. The IMF7 has the maximum correlation coefficient. It is a useful signal extraction for reconstruction. Take the IMF7 decomposition as the center and recombine the surrounding adjacent decompositions IMF6 and IMF8, which have a higher dependence on the original signal.

**Fig 8:**
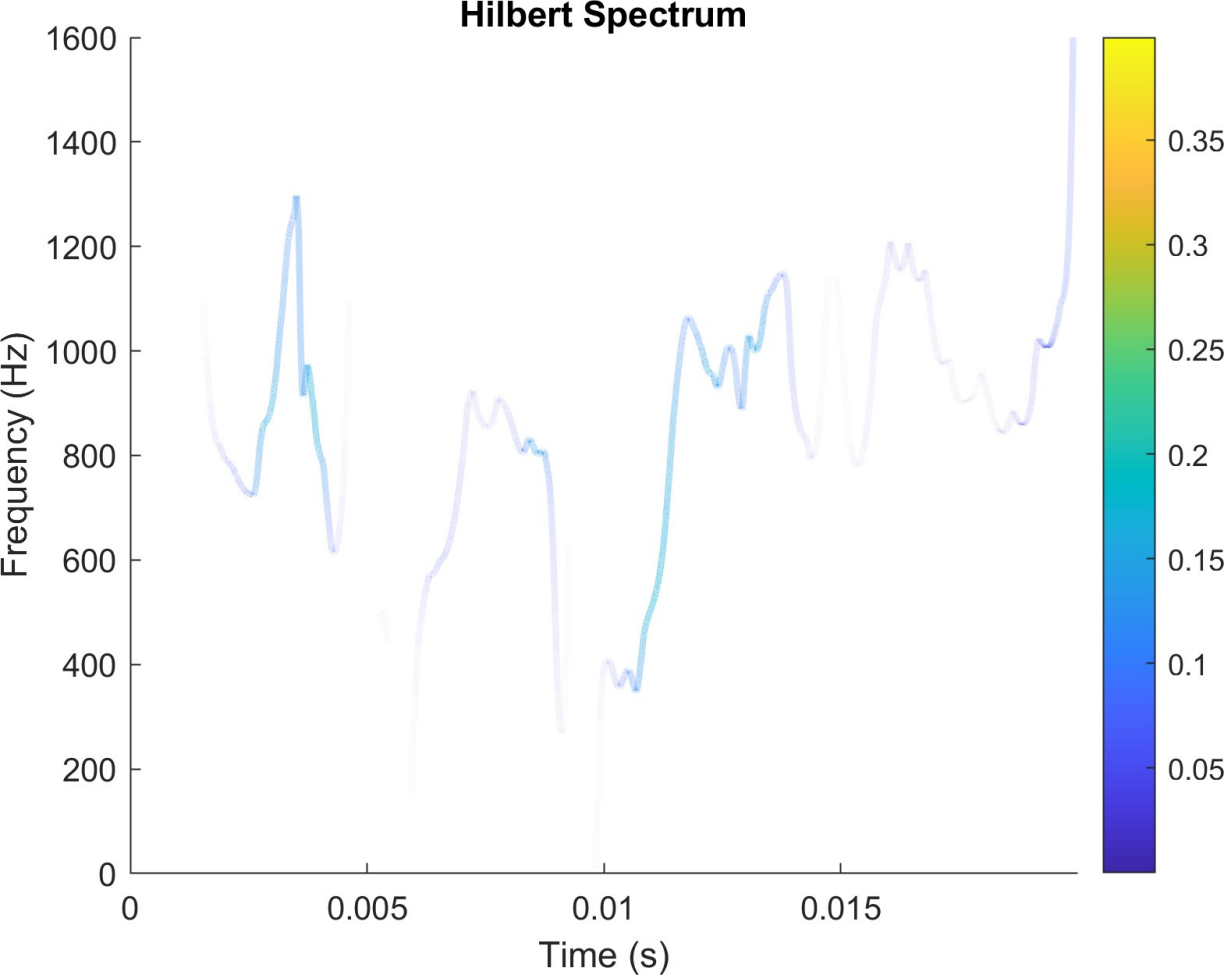
Hilbert spectrum of IMF7.

Hilbert spectrum is a time spectrum, which can be compared with the spectrum of continuous wavelet transform and short-time Fourier transform, which are also time-frequency analysis methods. This spectrum reflects the change of signal frequency components over time and is an important means to make non-stationary signals. Hilbert spectrum is made for IMF7, and the following results are obtained. With the gradual intensification, the amplitude of the Hilbert spectrum increases in the range of 800 ~ 1200Hz. The **hilbert spectrum of IMF7** is shown in Fig 8.

**Fig 9:**
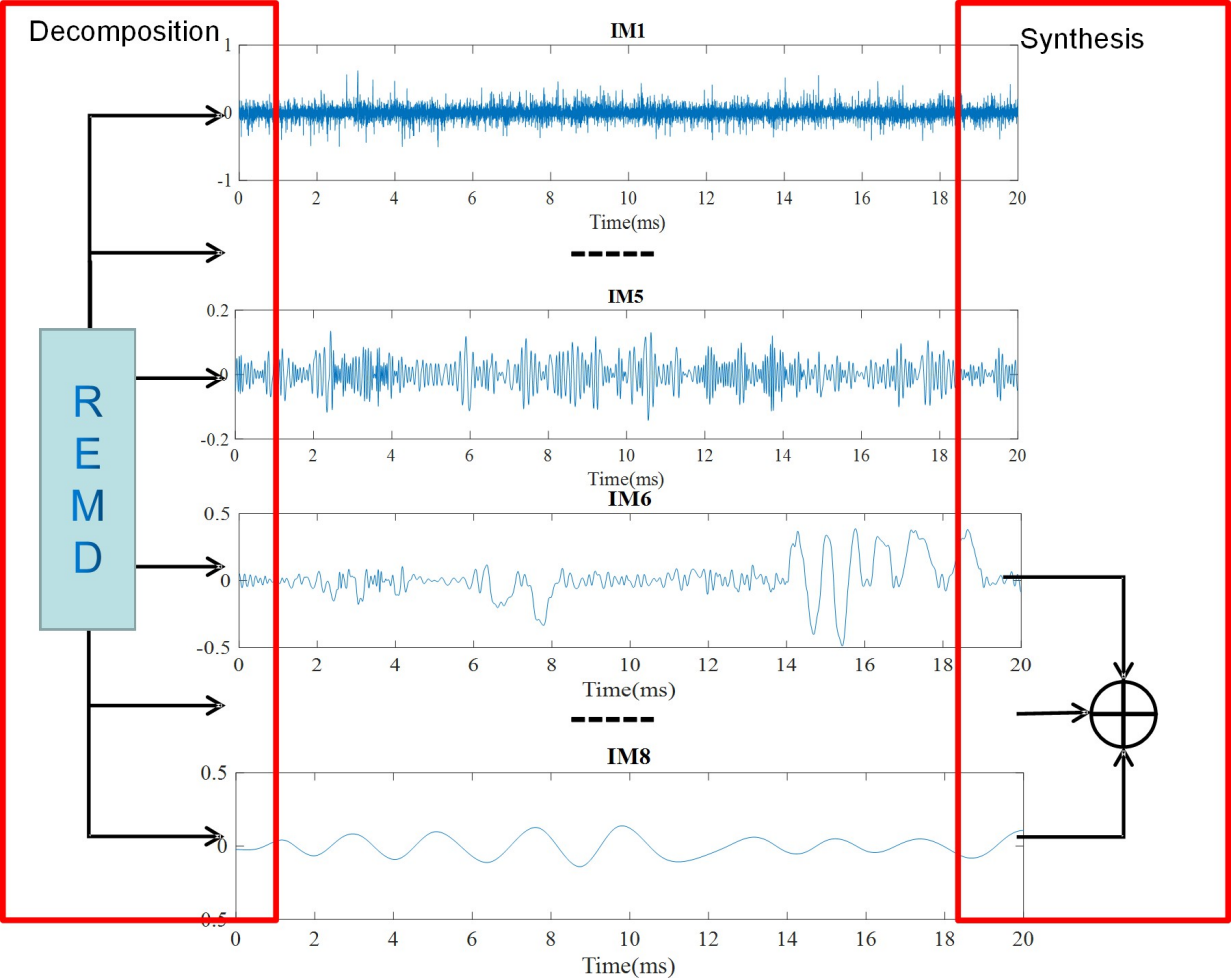
Decomposition and reconstruction process.

The corresponding decomposition and reconstruction processes are shown in Fig 9. Firstly, REMD decomposition is carried out. The REMD decomposition signal is composed of high-frequency signal and low-frequency signal. High-frequency signals are mostly noise signals deviate from the maximum correlation coefficient. And how to judge which part is a useful signal. It can be done by the correlation coefficient local capping between the obtained decomposed signal and the original signal. The position with the largest correlation coefficient is the most useful information. Due to the continuity of Doppler signal, the component adjacent to the maximum correlation coefficient is more important. By recombining these signals together, useful information with a high correlation with the doppler signal will can be obtained.

**Fig 10:**
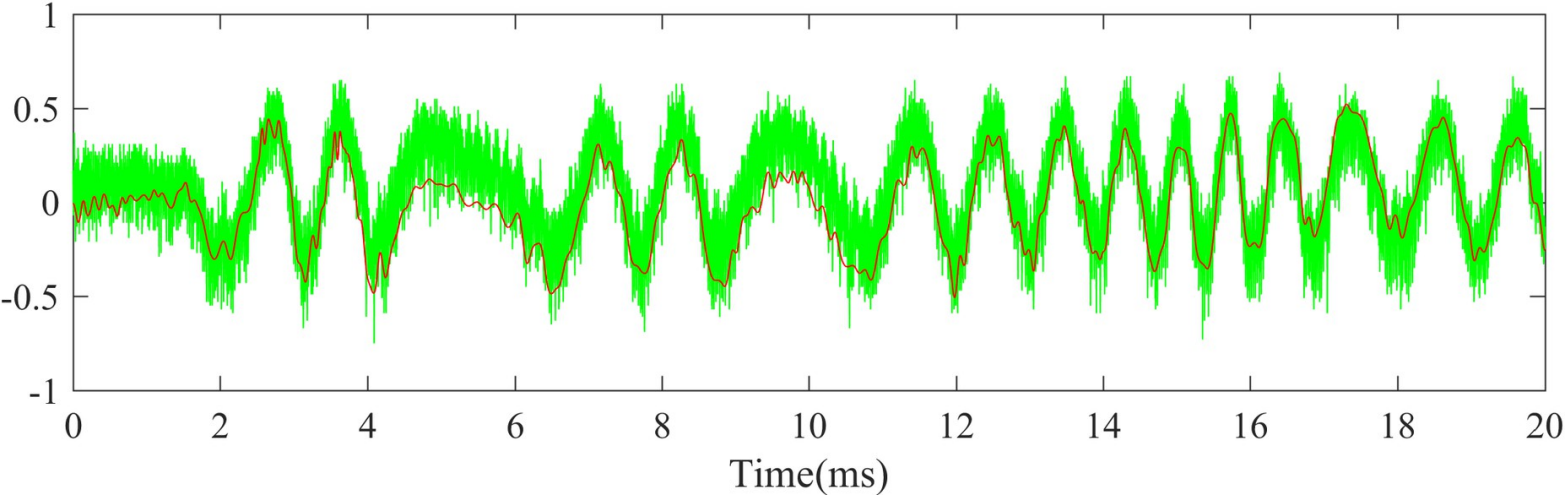
Comparison of denoised signal and the original signal.

The denoised signal and the original signal are shown in Fig 10. It can be seen that the high-frequency burring signal has been separated from the original signal. The denoised signal can better extract the relevant information in the original signal. Discrete wavelet decomposition could be used in this case, and the main difference is that there is no need to choose which wavelet to use in this method.

**Fig 11:**
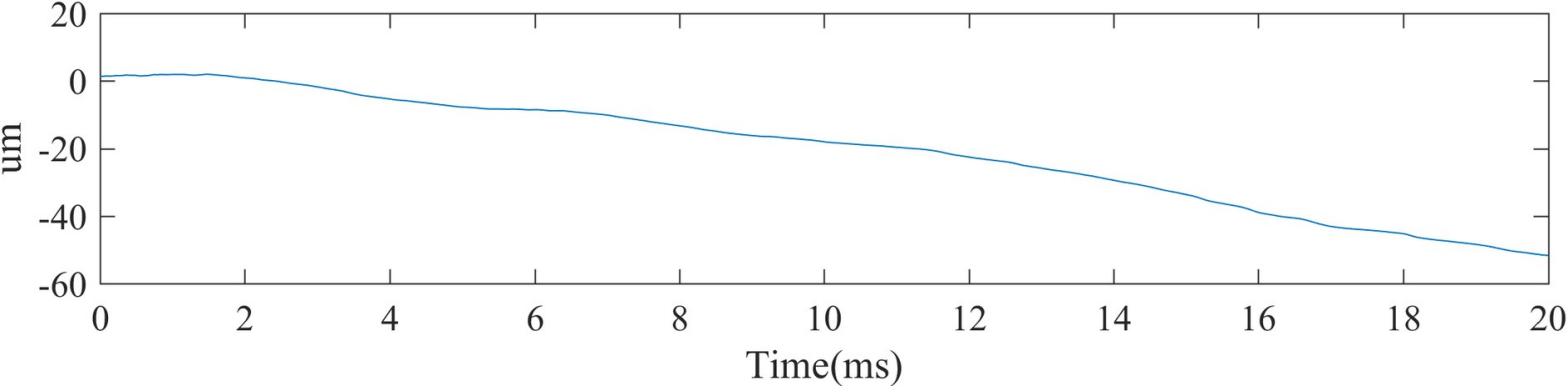
Displacement signal demodulated from noise-free signal.

The displacement signal can be obtained by solving the interference signal. The specific solution method is as follows. Firstly, the analytical signal of the signal is obtained by the Hilbert transform. The corresponding signal orthogonal to the original signal is obtained. This pair of signals is solved orthogonally. That is, arctangent transformation is used. The corresponding phase is extracted by arctangent transformation. Unwrap this phase item. Thus, the corresponding displacement is obtained. The displacement results are shown in Fig 11. The running speed of the guide rail carried by the stepper motor is set to 2.5 millimeter in per second, and the calculated data shown that the whole displacement is 50 micrometers within 20 milliseconds running time, and this result is in good agreement with the experimental setting.

## 4. Conclusion

The noise reduction process is completed using the correlation coefficient local capping REMD adaptive filter technique. There are three advantages:

1. IMFs decomposed by REMD contain high-frequency signals. These high-frequency signals which are deviate from the maximum correlation coefficients are discarded from the original signal.
2. With the highest correlation coefficient IMF and its local capping adjacent IMFs can be superimposed as reconstructed useful signals, which will get better analysis results, and extracts the low-frequency interference components in the signal.

This method is similar to the EWT method. The EWT method is divided into different frequency bands in the frequency domain. This method selects the point with the highest correlation coefficient as the center point of the useful signal according to the maxima distribution of the correlation coefficient, and expands it in a certain related field to obtain the useful combination of signals. Separate and remove the noise signal with high frequency. A correlation coefficient criterion for extraction useful intrinsic mode function (IMF) is proposed. This method is fully adaptive and suitable for the analysis of laser interference signals, and the code is freely available as open source on GitHub (https://github.com/awublack/local-capping-REMD).
